# Propofol total intravenous anaesthesia versus inhalational anaesthesia for acute postoperative pain in patients with morphine patient-controlled analgesia: a large-scale retrospective study with covariate adjustment

**DOI:** 10.1186/s12871-022-01683-9

**Published:** 2022-05-10

**Authors:** Stanley Sau Ching Wong, Edward Kwok Yiu Choi, Wing Shing Chan, Chi Wai Cheung

**Affiliations:** 1 Laboratory and Clinical Research Institute for Pain, Department of Anaesthesiology, The University of Hong Kong, HKSAR, Hong Kong, China; 2 Department of Anaesthesiology, Li Ka Shing Faculty of Medicine, The University of Hong Kong, HKSAR, Hong Kong, China

**Keywords:** Propofol, Total intravenous anaesthesia, Postoperative pain, Opioid consumption, General anaesthesia, Postoperative analgesia

## Abstract

**Background:**

To compare the postoperative analgesic effect of propofol total intravenous anaesthesia (TIVA) versus inhalational anaesthesia (GAS) in patients using morphine patient-controlled analgesia (PCA).

**Methods:**

A retrospective cohort study was performed in a single tertiary university hospital. Adult patients who used PCA morphine after general anaesthesia across 15 types of surgeries were included. Patients who received propofol TIVA were compared to those who had inhalational anaesthesia. Primary outcomes assessed were postoperative numerical rating scale (NRS) pain scores and postoperative opioid consumption.

**Results:**

Data from 4202 patients were analysed. The overall adjusted NRS pain scores were significantly lower in patients who received propofol TIVA at rest (GEE: β estimate of the mean on a 0 to 10 scale = -0.56, 95% CI = (-0.74 to -0.38), *p* < 0.001; GAS as reference group) and with movement (β estimate = -0.89, 95% CI = (-1.1 to -0.69), *p* < 0.001) from postoperative days (POD) 1–3. Propofol TIVA was associated with lower overall adjusted postoperative morphine consumption (β estimate = -3.45, 95% CI = (-4.46 to -2.44), *p* < 0.001). Patients with propofol TIVA had lower adjusted NRS pain scores with movement for hepatobiliary/pancreatic (*p* < 0.001), upper gastrointestinal (*p* < 0.001) and urological surgeries (*p* = 0.005); and less adjusted postoperative morphine consumption for hepatobiliary/pancreatic (*p* < 0.001), upper gastrointestinal (*p* = 0.006) and urological surgeries (*p* = 0.002). There were no differences for other types of surgeries.

**Conclusion:**

Propofol TIVA was associated with statistically significant, but small reduction in pain scores and opioid consumption in patients using PCA morphine. Subgroup analysis suggests clinically meaningful analgesia possibly for hepatobiliary/pancreatic and upper gastrointestinal surgeries.

**Trial registration:**

This study is registered at ClinicalTrials.gov (NCT03875872).

**Supplementary Information:**

The online version contains supplementary material available at 10.1186/s12871-022-01683-9.

## Background

Acute postoperative pain remains an important clinical problem [1]. Suboptimal postoperative pain control is associated with worse outcomes including reduced patient satisfaction, delayed recovery, development of chronic post-surgical pain, and increased morbidity [2–4]. It is important to come up with modalities that can improve postoperative analgesia.

The role of propofol total intravenous anaesthesia (TIVA) for acute postoperative pain is still unclear [5]. Some clinical studies have shown analgesic benefit [6–13], while others have shown no difference [14–17]. The analgesic effect of propofol TIVA may be influenced by a number of factors, including the choice of postoperative analgesic techniques. Intravenous patient-controlled analgesia (PCA) with opioid is an effective and commonly used method to deliver potent opioids quickly upon patient demand and improve postoperative analgesia [18, 19]. However, the analgesic effect of propofol TIVA in patients using PCA morphine has not been addressed.

In this study, we performed a large-scale retrospective cohort study to compare the acute postoperative analgesic effect of propofol TIVA versus inhalational anaesthesia in patients using PCA morphine. We studied the analgesic effect across 15 types of surgeries, including gynaecological, hepatobiliary and pancreatic, colorectal, upper gastrointestinal (oesophageal and gastric), head and neck, plastic and reconstructive, breast, limb, urological, trauma, oral and maxillofacial, endocrine, vascular, and spine surgeries. This allowed us to evaluate propofol TIVA’s analgesic effect in different surgeries.

## Methods

This was a retrospective cohort study. The study was approved by the Institutional Review Board of the University of Hong Kong/Hospital Authority Hong Kong West Cluster (UW 19–182) and registered at ClinicalTrials.gov (NCT03875872). This study was performed in accordance with the Declaration of Helsinki and the Strengthening the Reporting of Observational studies in Epidemiology (STROBE) checklist. The need for informed consent from patients was waived by the Institutional Review Board of the University of Hong Kong/Hospital Authority Hong Kong West Cluster. All of the data used for analysis were retrieved from the acute pain service database in Queen Mary Hospital, which is a tertiary university hospital. Records of patients with surgical operations performed from 1^st^ January 2015 to 30^th^ December 2017 were reviewed and analysed. Data collected included patient’s demographic data (age, body weight, gender, American Society of Anaesthesiologists (ASA) physical status), basic clinical data (e.g. patient diagnosis, surgical procedure performed, and medical diseases), type of surgical procedure, anaesthetic technique (TIVA with propofol or inhalational anaesthesia), intraoperative analgesics, postoperative numerical rating scale (NRS) pain scores, postoperative patient controlled analgesia (PCA) morphine consumption, postoperative analgesics and adverse events.

The precise general anaesthetic technique and drug dosage was provided at the discretion of the attending anaesthetist. Patients who received propofol TIVA (TIVA group) were induced and maintained with total intravenous propofol using the Marsh effect site model (Fresenius Kabi). Patients given inhalational anaesthesia (GAS group) were induced with an intravenous bolus of propofol followed by maintenance with sevoflurane or desflurane. In all patients, airway was secured by endotracheal intubation or insertion of a laryngeal mask airway. Nitrous oxide was not used in our hospital. Fentanyl or remifentanil was also used for induction. Muscle relaxation, if required, was achieved with rocuronium, atracurium or cisatracurium. The choice of intraoperative and postoperative analgesic drugs was given at the discretion of the anaesthetist. Intraoperative analgesic drugs that could be used included fentanyl, remifentanil, morphine, ketamine, dexmedetomidine, non-steroidal anti-inflammatory drugs (NSAIDs), paracetamol, intravenous lignocaine, and local wound infiltration with levobupivacaine. Postoperative analgesic drugs that could be prescribed included paracetamol, NSAIDs, tramadol, dihydrocodeine, pregabalin, gabapentin, amitriptyline, and morphine. Morphine was the only opioid that was used for PCA. Reversal of muscle relaxation after operation was achieved with neostigmine and atropine if required.

Only records of cases performed under general anaesthesia (propofol TIVA or inhalational anaesthesia) were included. In addition, we only included patients who used PCA morphine (morphine was the only opioid used for PCA in the hospital). Patients using other postoperative regional analgesic techniques such as epidural or peripheral nerve catheters were excluded. These other analgesic techniques were rarely used together with general anaesthesia in our hospital. Postoperative outcomes were compared between patients who received propofol TIVA to those who received inhalational anaesthesia. The primary outcomes that were assessed were postoperative NRS pain scores (at rest and with movement) and postoperative PCA morphine consumption. Secondary outcomes evaluated were the incidence of postoperative adverse effects. The type of adverse effects assessed were: nausea, vomiting, dizziness, and pruritus. We also recorded the number of patients who experienced postoperative confusion. Information about NRS pain scores were collected once a day during the acute pain round. Patients reported their pain scores at rest and with movement during assessment to the pain physician. Pain score was measured using a 0–10 scale, where 0 represented no pain and 10 represented the worse possible pain. Daily cumulative postoperative PCA morphine consumption (morphine consumed during the past 24 h) was recorded. Postoperative NRS pain scores and PCA morphine consumption were recorded for postoperative days (POD) 1, 2 and 3.

Potential predictors for postoperative pain used in the Generalized Estimating Equation (GEE) model included time, age, sex, body weight, ASA status, postoperative mechanical ventilation, chronic opioid/sedative user and type of surgery. Intraoperative and postoperative analgesic drugs were included as control variables to adjust for possible confounding factors in the statistical comparison of postsurgical outcomes. The type of intraoperative analgesic medications used were obtained from the anaesthetic record. These drugs included opioids (remifentanil, morphine, fentanyl), ketamine, dexmedetomidine and non-steroidal anti-inflammatory drugs (NSAIDs)/paracetamol. The type of postoperative oral analgesic drugs given to the patients were recorded each day during the acute pain round by the pain physician. Oral analgesic drugs used included tramadol, dihydrocodeine, paracetamol, NSAIDs.

GEE model of postoperative outcomes using type of general anaesthesia (propofol TIVA versus inhalational anaesthesia) as categorical predictor were adjusted for by the predictors and control variables for predicting postoperative NRS pain scores and PCA morphine consumption. Similar analysis for postoperative adverse events were performed by Pearson Chi-square test and unadjusted odds ratio (OR) was calculated. The adjusted OR was calculated if the significant unadjusted OR was found. The GEE model of postoperative NRS pain scores at rest or with movement was adjusted by all of the above-mentioned independent predictors and control variables and also by cumulative PCA morphine consumption. The GEE model of cumulative PCA morphine consumption was adjusted by the predictors and control variables and further by NRS pain scores. Data were further broken down by individual type of surgery to evaluate whether the relative postoperative analgesic effects of propofol TIVA versus inhalational anaesthesia also applied to each specific type of surgery.

Patient baseline variables, intraoperative and postoperative analgesic drugs for the two groups (propofol TIVA and inhalational anaesthesia) were compared using independent-samples t test, Mann–Whitney U test or chi-square test. Chi-square test of independence with analgesic technique was also provided for each type of surgery. Postoperative NRS pain scores and PCA morphine consumption were adjusted by the predictors and control variables using the GEE model, which was used to adjust for postoperative NRS pain scores and PCA morphine consumption for the multiple observation time points from POD 1–3 by accounting for working correlation matrix. Bonferroni correlation adjustment was made when each type of surgery was going to conduct GEE model separately. All statistical analyses were performed using IBM SPSS Statistics for Windows, Version 26.0 (IBM Corp., Armonk. NY, USA computer software).

Cases where there was missing data regarding predictors described previously (e.g. body weight, ASA status) were excluded from the final analysis. Cases where there was missing control variable data described previously (e.g. intraoperative analgesic medication) were also excluded. Only cases with non-missing outcome data including postoperative pain scores and postoperative morphine consumption were included in the final GEE model. Each individual analysis was conducted with complete set of data for the dependent and independent variables. No imputation for variables with missing data was attempted. A minimum sample size of 500 was suggested for the GEE model [20].

## Results

**Fig. 1:**
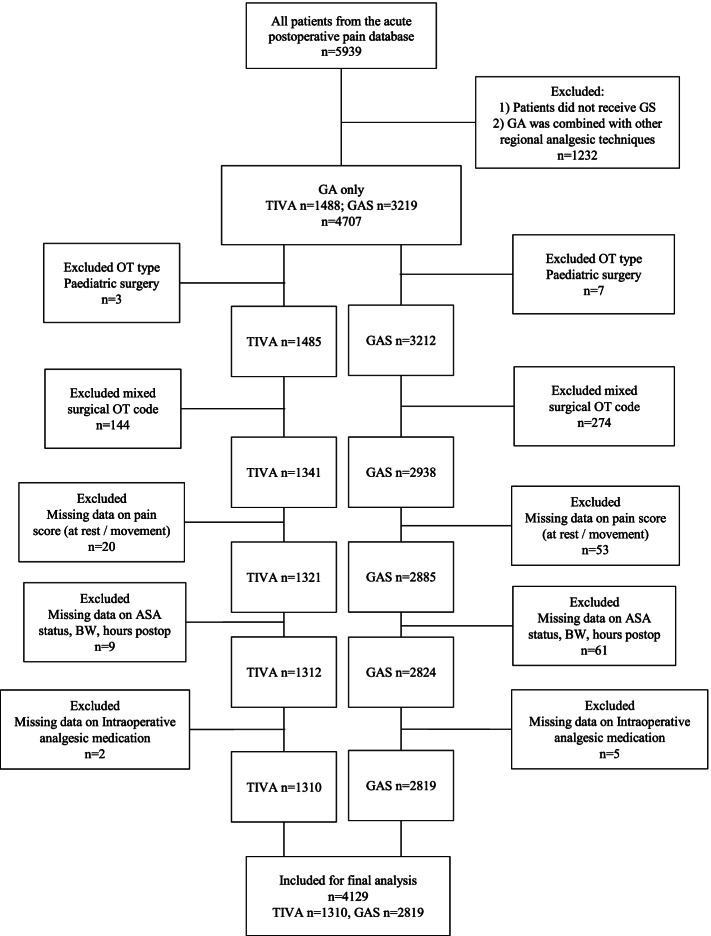
Patient flow diagram. GA indicates general anaesthesia; TIVA, total intravenous anaesthesia; GAS, inhalational anaesthesia; OT, operation; ASA, American Society of Anesthesiologists; BW, body weight

Five thousand nine hundred and thirty-nine surgical cases from 1^st^ January 2015 to 30^th^ December 2017 were screened from the acute pain service database (Fig. 1). Cases performed under neuraxial/regional anaesthesia or combined general-regional anaesthesia were excluded (*n* = 1232). Paediatric patients were excluded (*n* = 10). Surgeries with mixed type of operations were excluded (*n* = 418). Seventy-three cases without the information for NRS pain scores (at rest or with movement) were also excluded. Seventy cases were excluded because data on predictors such as ASA status, bodyweight, sex, age, number of hours postop were missing. Seven cases were excluded because data on intraoperative analgesic drugs were missing. After these exclusions, 4129 cases were included for final analysis. Two thousand eight hundred and nineteen cases were performed under inhalational anaesthesia and 1310 cases were performed under propofol TIVA.

Patients in the TIVA group were significantly younger, had lower overall ASA scores, had more females, and had fewer patients who required postoperative mechanical ventilation (all *p* < 0.05) (Table 1). There were no significant differences between the two groups in body weight or percentage of chronic opioid/sedative user (Table 1). Intraoperative dose of ketamine, remifentanil, morphine, and dexmedetomidine was significantly higher in the TIVA group (*p* < 0.001) (Table 1). Intraoperative fentanyl consumption was significantly higher in the GAS group (*p* < 0.001) (Table 1). The percentages of patients who received intraoperative NSAIDs/paracetamol and ‘other analgesics’ were significantly higher in the TIVA group (both *p* < 0.001) (Table 1). A significantly higher percentage of patients in the GAS group received postoperative tramadol (*p* < 0.001) (Table 1). There were no differences between the 2 groups in the use of other postoperative analgesic medication (Table 1). The distribution of patients amongst the different surgical procedures between TIVA and GAS group was significantly different (*p* < 0.001) (Table 1). Test of independence between each surgery and anaesthetic technique showed that a significantly higher percentage of patients received propofol TIVA in gynaecological, head and neck, upper gastrointestinal and breast surgeries (all *p* < 0.05) (Table 1). Inhalational anaesthesia was used significantly more frequently for hepatobiliary and pancreatic, colorectal, other abdominal and trauma surgeries (all *p* < 0.05).

**Table 1:** Patient characteristics, intra-operative analgesic drugs, postoperative analgesic drugs and type of surgical procedures

	GAS (*n* = 2819)	TIVA (*n* = 1310)	*p*-value
***Patient characteristics***			
Age, years	60.07 ± 15.64	58.23 ± 15.38	^a^ < 0.001
Sex			
* Male*	1487 (52.7%)	614 (46.9%)	^a^ < 0.001
* Female*	1332 (47.3%)	696 (53.1%)	
Body weight, kg	60.94 ± 13.19	60.79 ± 12.68	0.724
ASA			
* ASA I*	298 (10.6%)	160 (12.2%)	^a^ < 0.001
* ASA II*	1314 (46.6%)	676 (51.6%)	
* A SA III*	1140 (40.4%)	471 (36%)	
* ASA IV*	67 (2.4%)	3 (0.2%)	
Postoperative mechanical ventilation	99 (3.5%)	26 (2.0%)	^a^0.008
Chronic opioid/sedative user	21 (0.7%)	10 (0.8%)	0.949
***Intraoperative analgesics***			
Ketamine, mg	Mean = 15.62 Median = 15 IQR = 0–25	Mean = 24.08 Median = 20 IQR = 0–40	^a^ < 0.001
Morphine, mg	Mean = 7.11 Median = 7 IQR = 5–9	Mean = 8.64 Median = 8 IQR = 5–10	^a^ < 0.001
Remifentanil, µg	Mean = 1490.40 Median = 1200 IQR = 600–2000	Mean = 2960.49 Median = 2497.5 IQR = 1450–3926.25	^a^ < 0.001
Fentanyl, µg	Mean = 71.62 Median = 25 IQR = 0–100	Mean = 27.20 Median = 0 IQR = 0–0	^a^ < 0.001
Dexmedetomidine, µg	Mean = 3.02 Median = 0 IQR = 0–0	Mean = 10.40 Median = 0 IQR = 0–0	^a^ < 0.001
NSAIDS/Paracetamol, (yes/no)	833 (29.5%)	532 (40.6%)	^a^ < 0.001
Other analgesic drugs (yes/no)	1572 (55.8%)	926 (70.7%)	^a^ < 0.001
***Postoperative analgesics (yes/no)***			
Tramadol	1630 (57.8%)	594 (45.3%)	^a^ < 0.001
NSAID	71 (2.5%)	41 (3.1%)	0.261
Paracetamol	1636 (58%)	768 (58.6%)	0.72
Dihydrocodeine	563 (20%)	246 (18.8%)	0.369
Other analgesic drugs	1025 (36.4%)	468 (35.7%)	0.693
***Type of surgical procedures***			^a^ < 0.001
Hepatobiliary and Pancreatic	870 (30.9%)	268 (20.5%)	^a^ < 0.001
Colorectal	531 (18.8%)	123 (9.4%)	^a^ < 0.001
Gynaecological	350 (12.4%)	239 (18.2%)	^a^ < 0.001
Limb	240 (8.5%)	132 (10.1%)	0.103
Head and Neck	114 (4%)	192 (14.7%)	^a^ < 0.001
Urology	183 (6.5%)	87 (6.6%)	0.856
Spine	156 (5.5%)	68 (5.2%)	0.651
Upper gastrointestinal	108 (3.8%)	125 (9.5%)	^a^ < 0.001
Abdomen—Others	151 (5.4%)	6 (0.5%)	^a^ < 0.001
Breast	27 (1%)	27 (2.1%)	^a^0.004
Trauma	36 (1.3%)	5 (0.4%)	^a^0.007
Plastic and reconstructive	21 (0.7%)	12 (0.9%)	0.566
Vascular	22 (0.8%)	14 (1.1%)	0.354
Endocrine	6 (0.2%)	7 (0.5%)	0.131
Oral and Maxillofacial	4 (0.1%)	5 (0.4%)	0.153

^a^ significantly different at the 0.05 level

Values are mean ± SD or percentage (number) of patients; Kg indicates kilogram; *ASA* American Society of Anaesthesiologists physical status, *NSAID* non-steroidal anti-inflammatory drugs

The overall unadjusted postoperative NRS pain scores at rest were significantly lower in the TIVA group compared to the GAS group between POD 1–3 (β estimate = -0.5, 95% CI = (-0.68 to -0.32), *p* < 0.001; GAS as reference group) (Table 2). The β estimate implies that the mean pain score at rest in the TIVA group was 0.5 points lower than the GAS group. Patients in the TIVA group also had significantly lower overall unadjusted NRS pain scores with movement throughout the study period (β estimate = -0.9, 95% CI = (-1.1 to -0.7), *p* < 0.001; GAS as reference group) (Table 3).

**Table 2:** Postoperative pain scores (at rest): Difference between TIVA and GAS group

	n	POD 1	POD 2	POD 3	Group (GAS as reference) (Between-group comparison) β (95% CI of β)	*p*-value
Overall (unadjusted)					-0.50 (-0.68 to -0.32)	< 0.001
TIVA	1310	2.10 ± 2.02	1.48 ± 1.67	1.29 ± 1.56		
GAS	2819	2.48 ± 2.24	1.72 ± 1.86	1.44 ± 1.67		
^a^**Adjusted pain difference**						
Overall					-0.56 (-0.74 to -0.38)	< 0.001
TIVA	1310	2.10 ± 2.02	1.48 ± 1.67	1.29 ± 1.56		
GAS	2819	2.48 ± 2.24	1.72 ± 1.86	1.44 ± 1.67		
Hepatobiliary and Pancreatic					-0.67 (-1.06 to -0.28)	0.001
TIVA	268	2.00 ± 1.95	1.40 ± 1.62	1.19 ± 1.56		
GAS	870	2.51 ± 2.22	1.63 ± 1.78	1.30 ± 1.59		
Colorectal					-0.04 (-0.60 to 0.53)	0.902
TIVA	123	2.22 ± 2.10	1.28 ± 1.52	0.93 ± 1.18		
GAS	531	2.30 ± 2.17	1.53 ± 1.73	1.22 ± 1.52		
Gynaecological					-0.58 (-1.06 to -0.10)	0.018
TIVA	239	2.44 ± 2.16	1.65 ± 1.73	1.55 ± 1.65		
GAS	350	2.76 ± 2.16	1.87 ± 1.72	1.79 ± 1.70		
Limb					-0.64 (-1.24 to -0.05)	0.034
TIVA	132	1.87 ± 1.86	1.37 ± 1.56	1.29 ± 1.58		
GAS	240	2.50 ± 2.34	2.06 ± 2.15	1.76 ± 1.94		
Head and Neck					-0.17 (-0.80 to 0.47)	0.611
TIVA	192	2.21 ± 2.03	1.69 ± 1.78	1.45 ± 1.51		
GAS	114	2.41 ± 2.14	1.77 ± 1.69	1.77 ± 1.87		
Urology					-0.60 (-1.23 to 0.04)	0.064
TIVA	87	1.94 ± 1.88	1.17 ± 1.50	1.02 ± 1.40		
GAS	183	2.27 ± 2.18	1.48 ± 1.82	1.23 ± 1.56		
Spine					-0.47 (-1.39 to 0.44)	0.309
TIVA	68	2.51 ± 2.45	2.22 ± 2.23	1.96 ± 2.08		
GAS	156	2.91 ± 2.49	2.21 ± 2.15	1.85 ± 1.84		
Upper gastrointestinal					-0.42 (-1.04 to 0.21)	0.193
TIVA	125	1.70 ± 1.76	1.33 ± 1.54	1.10 ± 1.49		
GAS	108	1.96 ± 1.94	1.57 ± 1.69	1.16 ± 1.36		
Abdomen-Others					2.49 (-1.11 to 6.09)	0.176
TIVA	6	4.50 ± 3.08	1.00 ± 0.63	1.17 ± 0.98		
GAS	151	2.62 ± 2.54	1.75 ± 2.04	1.30 ± 1.69		
Breast					-0.84 (-2.09 to 0.41)	0.186
TIVA	27	1.30 ± 1.46	1.11 ± 1.63	1.07 ± 1.62		
GAS	27	1.93 ± 2.29	1.52 ± 1.91	1.33 ± 1.80

**Table 3:** Postoperative pain scores (with movement): Difference between TIVA and GAS group

	n	POD 1	POD 2	POD 3	Group (GAS as reference) (Between-group comparison) β (95% CI of β)	*p*-value
Overall (unadjusted)					-0.90 (-1.10 to -0.70)	< 0.001
TIVA	1310	4.85 ± 2.43	4.19 ± 2.19	3.93 ± 2.11		
GAS	2819	5.58 ± 2.42	4.78 ± 2.25	4.34 ± 2.16		
**#Adjusted pain difference**						
Overall					-0.89 (-1.10 to -0.69)	< 0.001
TIVA	1310	4.85 ± 2.43	4.19 ± 2.19	3.93 ± 2.11		
GAS	2819	5.58 ± 2.42	4.78 ± 2.25	4.34 ± 2.16		
Hepatobiliary and Pancreatic					-1.43 (-1.84 to -1.01)	< 0.001
TIVA	268	4.67 ± 2.17	4.28 ± 2.02	3.98 ± 1.97		
GAS	870	5.81 ± 2.36	4.94 ± 2.23	4.38 ± 2.14		
Colorectal					0.02 (-0.62 to 0.66)	0.958
TIVA	123	5.39 ± 2.47	4.20 ± 2.14	3.74 ± 1.86		
GAS	531	5.47 ± 2.47	4.58 ± 2.27	4.12 ± 2.12		
Gynaecological					-0.35 (-0.86 to 0.16)	0.182
TIVA	239	5.39 ± 2.40	4.49 ± 2.15	4.41 ± 2.11		
GAS	350	5.53 ± 2.22	4.55 ± 2.03	4.45 ± 1.99		
Limb					-0.36 (-1.07 to 0.35)	0.316
TIVA	132	4.82 ± 2.41	4.22 ± 2.13	3.96 ± 2.03		
GAS	240	5.41 ± 2.58	4.89 ± 2.47	4.61 ± 2.37		
Head and Neck					0.12 (-0.64 to 0.88)	0.755
TIVA	192	4.54 ± 2.36	3.77 ± 2.20	3.54 ± 2.10		
GAS	114	4.47 ± 2.60	3.80 ± 2.31	3.68 ± 2.46		
Urology					-1.11 (-1.88 to -0.34)	0.005
TIVA	87	4.91 ± 2.61	4.15 ± 2.31	4.01 ± 2.30		
GAS	183	5.62 ± 2.26	4.67 ± 2.07	4.25 ± 2.11		
Spine					-0.34 (-1.22 to 0.55)	0.456
TIVA	68	5.32 ± 2.63	5.04 ± 2.61	4.66 ± 2.36		
GAS	156	5.83 ± 2.59	5.31 ± 2.40	4.81 ± 2.13		
Upper gastrointestinal					-1.57 (-2.38 to -0.76)	< 0.001
TIVA	125	4.36 ± 2.44	3.95 ± 2.11	3.56 ± 2.05		
GAS	108	5.48 ± 2.25	4.86 ± 2.19	4.27 ± 2.13		
Abdomen-Others					0.62 (-2.67 to 3.90)	0.713
TIVA	6	6.50 ± 3.33	4.17 ± 2.64	4.17 ± 2.40		
GAS	151	5.95 ± 2.34	5.24 ± 2.09	4.36 ± 2.07		
Breast					-0.75 (-2.31 to 0.82)	0.351
TIVA	27	3.19 ± 2.30	3.00 ± 2.24	2.89 ± 2.08		
GAS	27	3.85 ± 2.51	3.81 ± 2.25	3.63 ± 2.27		

TIVA indicates total intravenous anaesthesia; GAS, inhalational anaesthesia; POD, postoperative day; mg, milli-grams; β, coefficient estimates; ^**^significantly different at the 0.05 level

^#^Analysis is adjusted by patient characteristics (time, age, gender, body weight, ASA, Postoperative ventilation, Chronic opioid/sedative user),

Intraoperative analgesics (Remifentanil (mcg), Fentanyl (mcg), Morphine (mg), Dexmedetomidine (mcg), Ketamine (mg), Had used NSAIDs or paracetamol),

Postoperative analgesics (Tramadol, NSAID, Paracetamol, Dihydrocodeine)

+ Type of surgical procedures: Trauma, Plastic and reconstructive, Vascular, Endocrine, Oral and Maxillofacial were not analysed separately due to the small sample size issue

The difference in NRS pain scores between TIVA group and GAS group was also adjusted by multiple predictors and control variables using the GEE model. TIVA group was associated with significantly lower overall adjusted NRS pain scores at rest between POD 1–3 (β estimate = -0.56, 95% CI = (-0.74 to -0.38), *p* < 0.001; GAS as reference group) (Table 2) and with movement between POD 1- 3 (β estimate = -0.89, 95% CI = (-1.1 to -0.69), *p* < 0.001; GAS as reference group) (Table 3).

The adjusted NRS pain scores at rest was significantly lower in the TIVA group for hepatobiliary and pancreatic surgery (β estimate = -0.67, 95% CI = (-1.06 to -0.28), *p* = 0.001; GAS as reference group) only (Table 2). The adjusted NRS pain scores with movement was significantly lower in the TIVA group for hepatobiliary and pancreatic surgery (β estimate = -1.43, 95% CI = (-1.84 to -1.01), *p* < 0.001; GAS as reference group), urological surgery (β estimate = -1.11, 95% CI = (-1.88 to -0.34), *p* = 0.005; GAS as reference group) and upper gastrointestinal surgery (β estimate = -1.57, 95% CI = (-2.38 to -0.76), *p* < 0.001; GAS as reference group) (Table 3). There were no significant differences in postoperative NRS pain scores between the 2 groups for other types of surgeries.

The overall unadjusted postoperative PCA morphine consumption was significantly lower in the TIVA group compared to the GAS group (β estimate = -3.41, 95% CI = (-4.41 to -2.41), *p* < 0.001; GAS as reference group) (Table 4). In addition, patients in the TIVA group had significantly lower adjusted PCA morphine consumption (β estimate = -3.45, 95% CI = (-4.46 to -2.44), *p* < 0.001; GAS as reference group) (Table 4). The adjusted PCA morphine consumption was significantly lower in the TIVA group for hepatobiliary and pancreatic surgery (β estimate = -4.29, 95% CI = (-6.48 to -2.1), *p* < 0.001; GAS as reference group), urological surgery (β estimate = -6.3, 95% CI = (-10.2 to -2.39), *p* = 0.002; GAS as reference group), and upper gastrointestinal surgery (β estimate = -7.41, 95% CI = (-12.66 to -2.17), *p* = 0.006; GAS as reference group) (Table 4).

**Table 4:** Postoperative morphine consumption: Difference between TIVA and GAS group

	n	POD 1	POD 2	POD 3	Group (GAS as reference) (Between-group comparison) β (95% CI of β)	*p*-value
Overall (unadjusted)					-3.41 (-4.41 to -2.41)	< 0.001
TIVA	1184	10.88 ± 10.40	8.17 ± 10.31	6.15 ± 8.44		
GAS	2606	13.67 ± 11.90	10.74 ± 11.66	7.83 ± 9.11		
**#Adjusted morphine consumption (mg)**						
Overall					-3.45 (-4.46 to -2.44)	< 0.001
TIVA	1184	10.88 ± 10.40	8.17 ± 10.31	6.15 ± 8.44		
GAS	2606	13.67 ± 11.90	10.74 ± 11.66	7.83 ± 9.11		
Hepatobiliary and Pancreatic					-4.29 (-6.48 to -2.10)	< 0.001
TIVA	240	11.34 ± 10.83	9.93 ± 11.40	7.00 ± 9.21		
GAS	787	14.55 ± 11.22	12.88 ± 11.79	8.70 ± 9.33		
Colorectal					-2.35 (-5.19 to 0.50)	0.107
TIVA	114	11.61 ± 9.82	6.87 ± 6.79	5.05 ± 6.31		
GAS	489	14.02 ± 11.92	10.79 ± 11.60	7.67 ± 8.75		
Gynaecological					-2.61 (-4.66 to -0.56)	0.013
TIVA	234	10.69 ± 9.10	5.55 ± 6.89	4.86 ± 6.06		
GAS	342	12.51 ± 9.28	6.51 ± 6.99	5.90 ± 5.89		
Limb					-0.01 (-2.83 to 2.81)	0.993
TIVA	130	8.35 ± 9.28	5.72 ± 8.48	4.48 ± 5.43		
GAS	230	10.24 ± 10.01	8.43 ± 10.38	7.14 ± 9.25		
Head and Neck					-2.97 (-6.61 to 0.67)	0.109
TIVA	185	11.88 ± 12.57	10.63 ± 12.56	7.97 ± 11.08		
GAS	106	13.93 ± 12.27	10.60 ± 13.72	8.23 ± 11.58		
Urology					-6.30 (-10.20 to -2.39)	0.002
TIVA	76	10.93 ± 9.13	8.05 ± 9.07	6.75 ± 8.76		
GAS	171	13.88 ± 13.44	9.77 ± 12.91	7.04 ± 9.39		
Spine					-2.48 (-6.71 to 1.75)	0.251
TIVA	63	9.84 ± 8.50	6.33 ± 8.85	4.75 ± 5.66		
GAS	149	11.85 ± 11.53	8.52 ± 11.12	6.90 ± 7.09		
Upper gastrointestinal					-7.41 (-12.66 to -2.17)	0.006
TIVA	75	11.55 ± 9.53	13.02 ± 13.24	8.10 ± 10.34		
GAS	90	16.12 ± 13.32	14.26 ± 14.03	10.06 ± 11.40		
Abdomen-Others					2.16 (-15.66 to 19.97)	0.812
TIVA	6	22.67 ± 22.84	5.83 ± 5.12	3.33 ± 3.67		
GAS	137	15.69 ± 15.23	14.01 ± 12.23	8.47 ± 9.31		
Breast					-1.65 (-5.48 to 2.17)	0.397
TIVA	27	4.82 ± 5.36	3.77 ± 6.78	3.99 ± 7.38		
GAS	26	6.60 ± 5.65	4.34 ± 4.45	3.91 ± 4.12		

TIVA indicates total intravenous anaesthesia; GAS, inhalational anaesthesia; POD, postoperative day; mg, milli-grams; β, coefficient estimates; ^**^significantly different at the 0.05 level

^#^Analysis is adjusted by patient characteristics (time, age, gender, body weight, ASA, Postoperative ventilation, Chronic opioid/sedative user),

Intraoperative analgesics (Remifentanil (mcg), Fentanyl (mcg), Morphine (mg), Dexmedetomidine (mcg), Ketamine (mg), Had used NSAIDs or paracetamol),

Postoperative analgesics (Tramadol, NSAID, Paracetamol, Dihydrocodeine)

+ Type of surgical procedures: Trauma, Plastic and reconstructive, Vascular, Endocrine, Oral and Maxillofacial were not analysed separately due to the small sample size issue

There were no differences in the overall unadjusted incidence of postoperative nausea (OR (Odds ratio) = 0.985, 95% CI = (0.833 to 1.165), *p* = 0.861, GAS as reference group), vomiting (OR = 1.224, 95% CI = (0.957 to 1.566), *p* = 0.108, GAS as reference group), dizziness (OR = 0.905, 95% CI = (0.76 to 1.078), *p* = 0.264, GAS as reference group) or pruritis (OR = 0.653, 95% CI = (0.422 to 1.01), *p* = 0.054, GAS as reference group) between the two groups (Tables 5).

**Table 5:** Odds ratio of the incidence of postoperative nausea, vomiting, dizziness and pruritis

	GAS (*n* = 2819)	TIVA (*n* = 1310)	OR (95% CI of OR)	*p*-value
Presence of Nausea	538 (19.1%)	247 (18.9%)	0.985 (0.833 to 1.165)	0.861
Presence of Vomiting	191 (6.8%)	107 (8.2%)	1.224 (0.957 to 1.566)	0.108
Presence of Dizziness	507 (18%)	217 (16.6%)	0.905 (0.760 to 1.078)	0.264
Presence of Pruritis	88 (3.1%)	27 (2.1%)	0.653 (0.422 to 1.010)	0.054

TIVA indicates total intravenous anaesthesia; GAS, inhalational anaesthesia; OR, odds ratio; GAS is the reference group for the odds ratio; % are unadjusted incidence of nausea, vomiting, dizziness or pruritis; ^**^significantly different at the 0.05 level

## Discussion

In this study, propofol TIVA was associated with overall reduction in NRS pain scores between POD 1–3 after surgery compared to inhalational anaesthesia in patients using PCA morphine. Propofol TIVA was also associated with lower postoperative morphine consumption. There were no differences in the overall incidence of nausea, vomiting, pruritus, and dizziness. A previous meta-analysis of randomized controlled trials found that propofol TIVA was associated with clinically significant, but small reduction in postoperative pain scores at 24 h after surgery compared to inhalational anaesthesia [12]. Another meta-analysis showed reduced postoperative pain scores and morphine consumption with propofol TIVA, but this was not clinically significant when a *p*-value of less than 0.01 was used to account for heterogeneity [17]. In a scoping review of randomized controlled trials that assessed postoperative analgesia as a primary outcome, propofol TIVA was associated with improved postoperative analgesia in 9 out of 16 clinical trials [21]. Our current study specifically investigated the analgesic effect of propofol TIVA in patients with PCA morphine.

Although there is a shift towards using more oral and regional analgesia, PCA opioids remains a commonly used analgesic technique [22]. A survey of over 17000 patients from an acute pain service found that 52% of patients used PCA opioids, while another survey found that PCA was used in 79% of hospitals in Germany [23, 24]. PCA opioid is recommended when parenteral opioids are required [25]. It is more effective than non-PCA opioid therapy in reducing postoperative pain and also results in higher patient satisfaction [18]. Since the analgesic effect of propofol TIVA is not large, its postoperative analgesic effect may be masked by the use of PCA opioid. Therefore, it is important to evaluate the analgesic efficacy of propofol TIVA when used in this context.

A significantly lower overall NRS pain score with movement and opioid consumption between POD 1–3 was found for hepatobiliary/pancreatic, urological, and upper gastrointestinal surgeries. Hepatobiliary/pancreatic surgery was also associated with reduced pain scores at rest. No differences were observed for other types of surgeries.

This suggests the analgesic effect of propofol TIVA was procedure specific. We also found that the overall difference in NRS pain scores between TIVA group and GAS group was small, being less than 1/10 for all time points both at rest and with movement. An NRS pain score of 1.3/10 or more has been associated with ‘minimal clinical improvement’ [26]. In our subgroup analysis, only hepatobiliary/pancreatic and upper gastrointestinal surgery had an NRS pain score improvement of more than 1.3/10 during movement. This suggests that the overall analgesic benefit of propofol TIVA may be limited, but is perhaps clinically meaningful for hepatobiliary/pancreatic and upper gastrointestinal surgeries. Patients undergoing hepatobiliary/pancreatic surgery may be more likely to have problems with drug metabolism, organ function, thrombocytopenia, and/or coagulopathy during the perioperative period. This could limit the use of analgesics such as paracetamol, NSAIDs, opioids and epidural analgesia, therefore making the analgesic effect of propofol TIVA more prominent. Another possible reason why a more significant difference was found with hepatobiliary/pancreatic surgery may be because the sample size was larger compared to other types of surgeries, therefore providing more statistical power to detect differences in outcome. Upper gastrointestinal surgeries produce upper abdominal wounds that typically lead to higher levels of postoperative pain, especially with movement such as coughing. Furthermore, oral analgesic medications are usually not allowed initially after upper gastrointestinal surgeries. These could explain the positive analgesic effect for this type of surgery.

We also evaluated the opioid sparing effects of propofol TIVA. Overall postoperative PCA morphine consumption was significantly lower for patients given propofol TIVA. Opioids are associated with adverse effects such as nausea, vomiting, sedation, ileus, pruritus, and respiratory depression. Minimizing opioid consumption is one of the main goals of multimodal analgesia, and can reduce adverse effects and improve patient outcomes [27]. PCA morphine consumption reduced by around 20%, 24% and 21% on POD 1, 2, and 3 respectively with propofol TIVA. It is unclear how much clinical benefit this amount of opioid reduction would produce.

Propofol is a short acting drug. However, it was associated with a relatively extended duration of analgesia (reduced pain scores and opioid consumption from POD 1–3). This suggests that propofol has preventive analgesic effects, which describes the phenomenon where the target drug reduces pain scores or analgesic consumption beyond its clinical duration of action (5 half-lives). One possible mechanism to explain this is propofol’s inhibitory effect on N-methyl-D-aspartate (NMDA) receptors [28, 29]. Inhibition of NMDA receptors using NMDA antagonists have been shown to produce preventive analgesic effects [30]. Another potential mechanism may be via propofol’s effect on the exchange protein directly activated by cAMP (EPAC). EPAC is involved in the transition from acute to persistent pain, and propofol has been shown to reduce spinal dorsal horn EPAC1 expression in an animal model for postoperative pain [31, 32].

We evaluated the effect of propofol TIVA on the incidence of adverse events, and did not find a significant overall difference for the incidence of nausea, vomiting, pruritus or dizziness. While propofol TIVA is known to reduce the incidence of nausea and vomiting, our results did not show any differences. This may be due to differences in local practice. Nitrous oxide is almost never used in our hospital where the study was conducted. In addition, patients are usually pre-emptively given prophylactic intraoperative anti-emetics such as dexamethasone and serotonin antagonist. These practices may have mitigated the anti-emetic benefit of propofol TIVA. The incidence of postoperative vomiting and nausea in this study was less than 10% and 20%, respectively, which is lower than the generally quoted incidence of 20–40% [33].

There were several limitations in this study. Data from this study was collected retrospectively, and may be prone to bias. We have statistically adjusted for confounders that were found to influence postoperative analgesic outcome. However, there were factors that could not be controlled. Since the acute pain database did not have information on chronic pain and pre-existing depression/anxiety, we were not able to control for these two potential confounding factors. We have controlled for the confounding factors that could be identified from the database, and the sample size (4129) in our study is much larger than the suggested minimum sample size (500). This would help reduce the estimated biases, since the estimated biases are smaller for larger sample sizes [20]. Another potential confounding factor that could not be controlled was depth of anaesthesia. However, there has been no recommendation advocating the routine use of depth of anaesthesia monitoring for every patient undergoing general anaesthesia [34], and this is not routinely monitored in our hospital. Depth of anaesthesia has not been shown by most clinical studies to affect postoperative pain scores, especially at 24 h or beyond [35–40]. Depth of anaesthesia is unlikely to be a significant confounding factor, especially since we studied postoperative analgesia from POD 1 to POD 3. A third limitation was that it was not possible to control for specific types of surgery within each type of surgical specialty. Another limitation was that while we had information about the type of oral postoperative analgesic drugs given, we did not know the precise dosage. Finally, while pain intensity such as NRS pain scores is the major outcome measure used for studying acute postoperative pain, one weakness is that it does not provide detailed information on functional and experiential aspects [41]. However, we have captured data on pain intensity with movement, which is a useful guide for functional impact [42].

## Conclusions

Propofol TIVA was associated with reduced acute postoperative pain and opioid consumption after surgery compared to inhalational anaesthesia in patients using PCA morphine. However, its analgesic effect was small and appears to be procedure specific. It may provide clinically meaningful pain reduction in hepatobiliary/pancreatic and upper gastrointestinal surgeries.

## Supplementary Information

**Additional file 1: **Dataset.

## Data Availability

All data generated or analysed during this study are included in this published article [and its supplementary information files].
